# Eco-friendly synthesis of ZnO, CuO, and ZnO/CuO nanoparticles using extract of spent *Pleurotus ostreatus* substrate, and their antioxidant and anticancer activities

**DOI:** 10.1186/s11671-025-04199-6

**Published:** 2025-02-13

**Authors:** Simangele Ngwenya, Nkanyiso J. Sithole, Khosi Ramachela, Doctor M. N. Mthiyane, Mulunda Mwanza, Moganavelli Singh, Damian C. Onwudiwe

**Affiliations:** 1 Crop Science Department, Faculty of Natural and Agricultural Science, North-West University, Mmabatho, 2035 South Africa; 2 Food Security and Safety Focus Area, Faculty of Natural and Agricultural Sciences, North-West University, Mmabatho, 2735 South Africa; 3 Department of Animal Science, Faculty of Natural and Agricultural Sciences, North-West University, Mmabatho, South Africa; 4 Department of Animal Health, Faculty of Natural and Agricultural Sciences, North-West University, Mmabatho, South Africa; 5 Nano-Gene and Drug Delivery Laboratory, Department of Biochemistry, University of KwaZulu-Natal, Durban, 4000 South Africa; 6 Material Science Innovation and Modelling (MaSIM), Faculty of Natural and Agricultural Sciences, North-West University, Mmabatho, South Africa

**Keywords:** Green synthesis, Metal oxides, Plant extracts, Biological assays, Biomedical

## Abstract

Biosynthesis techniques for nanomaterials have advanced significantly, promoting eco-friendly synthesis chemistry as a sustainable alternative to conventional methods. This study presents a novel and environmentally friendly approach for synthesizing nanoparticulate ZnO, CuO, and ZnO/CuO nanocomposites using aqueous extracts of *Pleurotus ostreatus* spent substrate, is reported. The structural, optical, and morphological properties of the synthesized NPs were analysed. A hexagonal phase of ZnO NPs and a monoclinic phase of CuO NPs were obtained according to the X-ray diffraction analysis. A reduction in the peak intensity of these metal oxides was observed in the ZnO/CuO NPs due to reduced crystallinity. The absorption spectra, obtained from the UV–vis analysis, showed peaks at 354, 365, and 525 nm for the ZnO, CuO, and ZnO/CuO NPs, respectively. An anticancer assay of the NPs was conducted using human embryonic kidney (HEK 293) and cervical carcinoma (HeLa) cell lines, while a 1,1-diphenyl-2-picrylhydrazyl (DPPH) assay was used for the antioxidant evaluation. The ZnO, CuO, and ZnO/CuO NPs showed higher antioxidant potency with IC_50_ of 2.15, 2.16, and 3.18 µg/mL, respectively, than the ascorbic acid (4.25 µg/mL). This indicates that the nanoparticles were more effective in capturing DPPH free radicals. Anticancer assays showed strong cytotoxic effects for all nanoparticles, with ZnO NPs exhibiting the highest activity (IC_50_: 1.94 μM for HEK 293 cells, 3.23 μM for HeLa cells), surpassing CuO and ZnO/CuO NPs. Cell viability for both HEK 293 and HeLa cells decreased as nanoparticle concentration increased, confirming dose-dependent cytotoxicity. The green synthesized metal oxides and their composite have the potential for biomedical applications.

## Introduction

Zinc oxide (ZnO) and copper oxide (CuO) nanoparticles have attracted considerable interest recently due to their exceptional chemical stability (under different conditions), affordability, and large surface area. These nanoparticles hold great potential for a wide array of biomedical applications, such as medical diagnostics, drug delivery, antibacterial treatments, antioxidant applications, anti-inflammatory therapies, and anticancer activities [1, 2]. Nanoparticles interact with malignant cells via unique physicochemical properties, enabling targeted therapy. Their small size and high surface area allow efficient cellular penetration. By generating reactive oxygen species (ROS) through interactions with cellular components or catalytic activity, they disrupt mitochondrial function, damaging membranes, proteins, and DNA to induce apoptosis. Engineered nanoparticles can also neutralize ROS near healthy DNA, acting as antioxidants or scavengers, minimizing harm to normal cells while maximizing cytotoxic effects on cancer cells [3]. The therapeutic effects of nanoparticles are influenced by factors such as particle size, the duration of the target cell culture, the metal content within the target cell, and their physicochemical properties [4–6]. Furthermore, bimetallic and multimetallic nanoparticles, which contain two or more metals within the entity either as complex metal oxides, doped metal oxides, or nanocomposites (for example ZnO/CuO NPs), exhibit distinctive physicochemical properties with synergistic effects and enhanced functionality [7]. These combined nanoparticles offer more reactive sites, improved efficiency, and greater stability [8]. The improved properties are attributed to the incorporation of different metals into an oxide matrix, resulting in the creation of materials with enhanced chemical and physical properties. In a composite of ZnO and CuO NPs, with compatible band potentials within an oxide matrix, the isolated entity can exhibit modified properties due to the interactions between the individual metals and oxygen [9]. This significantly enhances the performance of the nanocomposite by facilitating charge transfer, improving charge separation efficiency, and extending the lifetime of carriers at this nanostructured scale [10].

Although various chemical and physical methods have been used to prepare ZnO and CuO nanoparticles, they present significant challenges. These include using toxic reagents, complex synthesis processes, the requirement for specialised equipment, high consumption of energy, and high costs. These factors make large-scale applications of these metal oxides impractical, particularly in cosmetics, pharmaceuticals, food, and environmental sectors. An effective solution to these issues is the green synthesis of nanoparticles, which offers a promising alternative [11–13]. This eco-friendly method for the synthesis of metal oxide nanoparticles involves the use of biomaterials and metabolites from plants, algae, fungi, mushrooms, and truffles [14, 15]. Among these biological routes, the use of plant extract is more favourable due to its ease of handling, availability, and compatibility with the intended biomedical applications such as antibacterial and antifungal agents, drug delivery, anti-oxidant, and cancer treatment [16]. The use of plant extract in green synthesis eliminates the necessity for chemical stabilizing agents to maintain nanoparticle size and circumvents the need for toxic chemical reactants such as reducing or oxidizing agents [17]. The biomolecules extracted from plants contain natural substances such as vitamins, enzymes, and amino acids, that are useful for the bio-synthesis of nanoparticles for various applications [18–20].

Various plants have been employed in the synthesis of different metal oxide nanoparticles [21–24]. Yadav et al., reported the biosynthesis of ZnO and CuO nanoparticles using *Ficus benghalensis* extracts [25]. Pomegranate (*Punica granatum*) extract was reported for the biosynthesis of ZnO and CuO nanoparticles [26]. The extracts of *Rumex crispus* seeds and leaf extract of *Aloe barbadensis* miller (Aloe vera) have been utilized to mediate the synthesis of CuO nanoparticles [27, 28].[23] *Argyreia nervosa* leaf extract was used in a simple and environmentally friendly route to prepare ZnO, CuO, and CuO-ZnO nanomaterials [29]. Extract of *Calotropis gigantea* leaf has also been used to synthesize ZnO/CuO nanocomposite by adopting the solution combustion method [30]. Bekru et al., synthesized CuO/ZnO nanocomposites using an aqueous extract obtained from *Verbascum sinaiticum* Benth [31]. *The* phytochemical compounds obtained from parsley leaf extract have been reacted with the precursor salts for ZnO and CuO nanoparticles, resulting in the formation of ZnO/CuO nanocrystals [32].

In this paper, we describe a simple green synthesis of ZnO, CuO, and ZnO/CuO nanoparticles mediated by the aqueous extract of *Pleurotus ostreatus* mushroom. *Pleurotus ostreatus* is rich in bioactive compounds with therapeutic potential, including polysaccharides, flavonoids, terpenoids, phenolics, steroids, glycoproteins, ergothioneine, and beta-carotene [33, 34]. These phytocomponent species could mediate nanoparticle formation by reducing the metal salts to their nanoparticulate scale [35–38]. The optical properties, phase structure, and morphology were analysed using UV–vis spectroscopy, X-ray diffraction (XRD), scanning electron microscopy (SEM), and transmission electron microscopy (TEM). Furthermore, the anticancer activities of the synthesized materials were evaluated against human embryonic kidney (HEK 293) and cervical carcinoma (HeLa) cell lines. The antioxidant properties were assessed using the 1,1-diphenyl-2-picrylhydrazyl (DPPH) assay. The results demonstrated that the samples exhibited the potential to inhibit the growth of the tested cell lines and act as antioxidants.

## Materials and methods

### Materials

*Pleurotus ostreatus* spawns, perforated plastic mushroom substrate bags, and mushroom biofortificants were purchased from Eco-Agro Enterprise (Pty) Ltd in Mpumalanga Province. Sunflower husks were sourced from the Kgora farmer training centre in Mahikeng. Zinc acetate dihydrate [Zn(CH_3_CO_2_)_2_·2H_2_O], copper(II) acetate [Cu(CO_2_CH_3_)_2_], sodium hydroxide (NaOH), and all other chemicals were purchased from Merck (Pty) Ltd. Sterile plastic ware for cell culture was obtained from Corning Inc. (New York, NY, USA). Throughout the experiment, an ultrapure (18 MOhm) water (Millipore, France) was utilized.

### Preparation of spent *Pleurotus ostreatus* mushroom

The *P. ostreatus* mushroom substrate preparation and the inoculation process were carried out following the procedure reported by Mhlongo, Mnisi [39]. Sunflower husks (10 kg) were moistened with 10 L of distilled water overnight. Thereafter, they were sterilized by steaming at 100 °C for 1 h using the method described by Tuyen, Phuong [40]. The sterilized substrate was then cooled to room temperature (RT) and inoculated with spawn at a concentration of 500 g/kg in plastic mushroom bags replicated six times [39, 41]. The inoculated substrate replicates were kept in the dark at RT and occasionally sprinkled with water to maintain humidity and moisture. On day 28 post-inoculation, the developed mycelia were removed manually, and the spent mushroom substrate (SMS) was dried under shade until constant weight. The dried SMS was ground to pass through a 1 mm sieve (Polymix PX-MFC 90 D, Switzerland) and then stored for future use.

### Extraction of spent *Pleurotus ostreatus* mushroom substrate

About 10 g of the dried SMS was added to 100 mL of de-ionized water, heated at 70 °C, and stirred for 2 h. Thereafter, the mixture was allowed to cool to room temperature, filtered, and the filtrate was stored in the refrigerator for further experiment.

### Synthesis of ZnO NPs

**Fig. 1:**
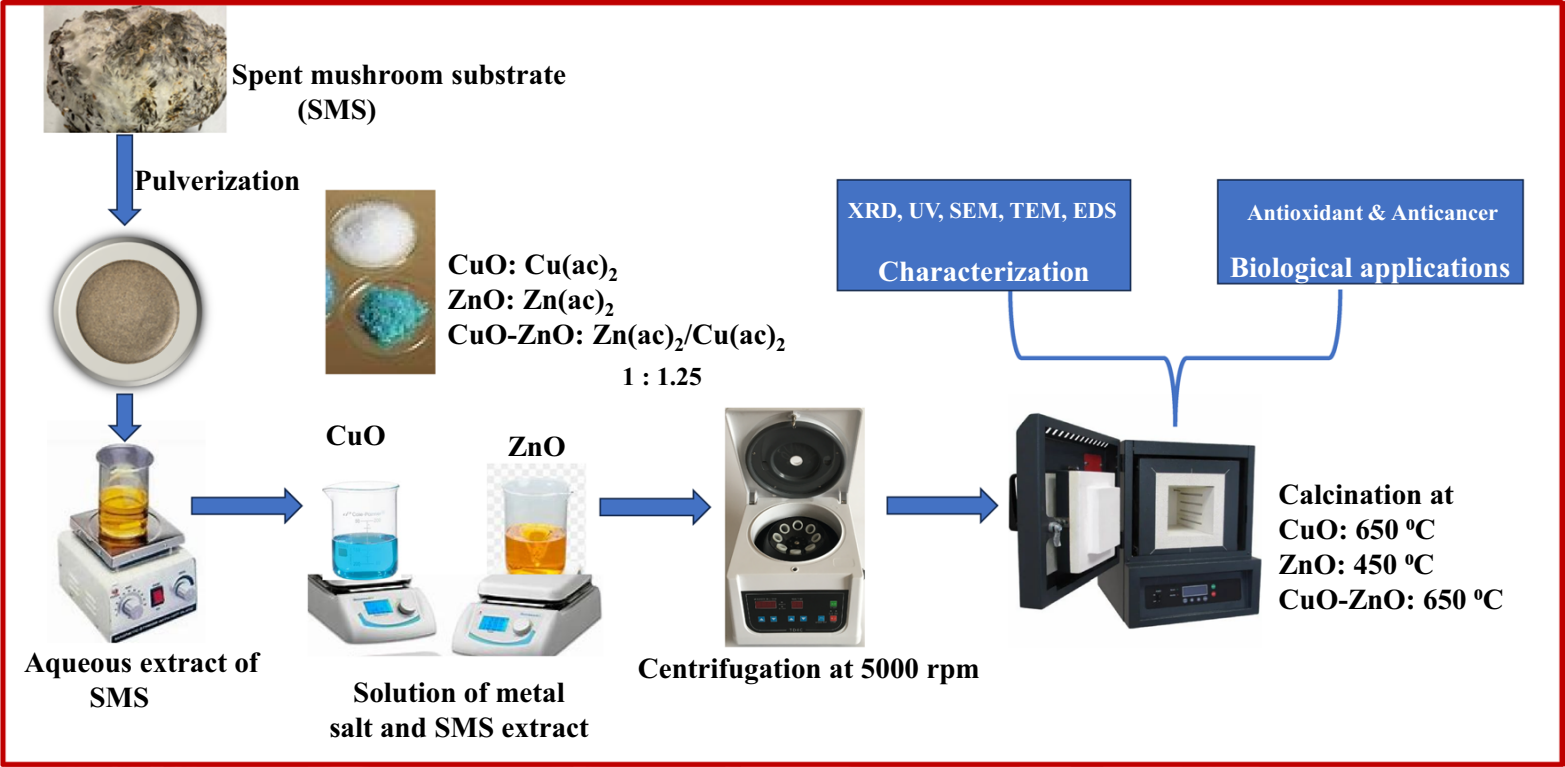
Scheme depicting the synthesis, characterization and biological applications of CuO, ZnO and ZnO/CuO NPs

Approximately 2 g of zinc acetate, Zn(CH_3_CO_2_)_2_·2H_2_O, was dissolved in 20 mL aqueous extract of *Pleurotus ostreatus* spent mushroom substrate. The pH of the solution was adjusted to 8 using sodium hydroxide (NaOH), heated up to 80 °C and then maintained at this temperature until the formation of a deep yellow colouration. The suspension formed was then centrifuged at 5000 rpm for 10 min, rinsed with distilled water three times followed by ethanol to remove the impurities. The obtained solid was oven dried at 80 °C for 5 h and then calcinated in a furnace at 450 °C for 2 h (Fig. 1) [42].

### Synthesis of CuO NPs

Following the similar method reported for the synthesis of ZnO NPs, 2.5 g of copper(II) acetate was dissolved in 20 mL of distilled water. While stirring this solution, 20 mL of the spent mushroom was gradually added, and the temperature was maintained at 80 °C for 2 h. After the reaction, the dark-coloured green solution was cooled to R.T and then centrifuged 3 times with water and ethanol. The solid product was collected in a ceramic crucible, dried in oven for 6 h, and then calcinated at 650 °C for 2 h [43].

### Synthesis of ZnO/CuO NPs

The ZnO/CuO NPs was synthesised by mixing a solution of 2.5 g of copper acetate and 2 g of zinc acetate in 30 mL of distilled water. Then 30 mL extract of the *P. ostreatus* spent mushroom substrate was added and the solution pH was adjusted to 8 using NaOH and heated up to 80 °C for 2 h. The solution was centrifuged after the reaction, dried overnight in an oven and calcinated at 650 °C for 2 h (Fig. 1).

### Characterization of the NPs

Structural phase identifications of the synthesized nanomaterials were analysed using Bruker D8 Advanced X-ray diffraction (XRD) instrument (Karlsruhe, Germany). A TECNAI G2 (ACI) Transmission electron microscopy (TEM) equipment (Hillsboro, OR, USA), with an accelerating voltage of 200 kV, and FEI Quanta FEG 250 Scanning electron microscopy (SEM) were used for the morphological analysis. Elemental composition was determined using Energy-dispersive X-ray (EDX) spectroscopy, whilst the optical measurement was carried out using a Perkin Elmer Lambda 20 UV–vis spectrophotometer.

### Anticancer evaluation of the nanoparticles

A cytotoxicity assay was conducted following a modified protocol by Adeyemi et al. [65]. Human embryonic kidney (HEK 293) and cervical carcinoma (HeLa) cell lines from ATCC were cultured in EMEM supplemented with 10% fetal bovine serum, penicillin, and streptomycin. Cell viability was assessed using the MTT assay. Cells were seeded in a 96-well plate at 2.5 × 10^2^ cells/well and incubated overnight at 37 °C. Test samples (10–100 µg) were added, and cells were incubated for 48 h. MTT reagent was then added, followed by a 4-h incubation. Formazan crystals were dissolved in DMSO, and absorbance was measured at 570 nm using a microplate reader. DMSO served as the blank, with untreated cells and 5-Fluorouracil as controls. Experiments were conducted in triplicate to ensure reliability.

### Statistical analysis

Statistical analysis was performed using an analysis of variance (ANOVA) tool on the data collected. Tukey’s test was applied to identify significant differences in mean values where ANOVA indicated significance (P < 0.05). Most statistical calculations were conducted with Origin Lab software, with each experiment and assay replicated three times (n = γ). The results are reported as the mean of these three values, expressed as mean ± standard deviation.

## Results and discussion

### The optical properties

**Fig. 2:**
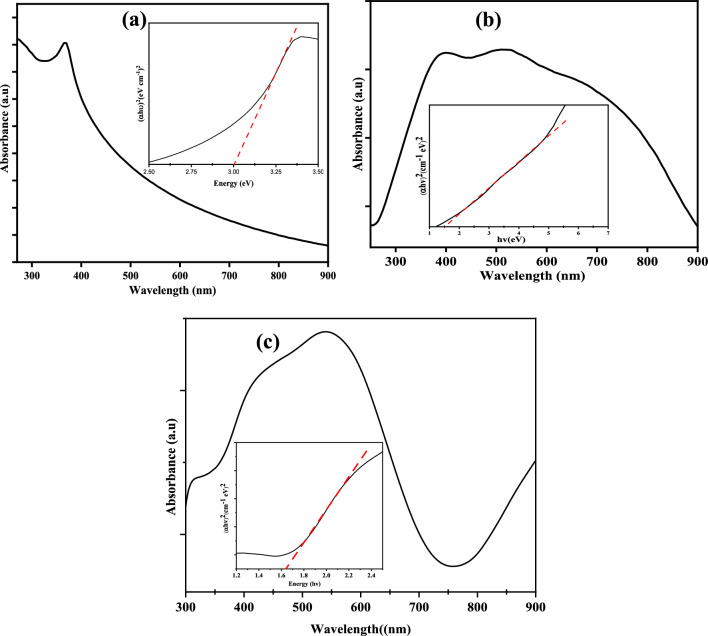
UV visible spectra of **a** ZnO, **b** CuO, **c** ZnO/CuO nanoparticles synthesized using extract of SMS; and their corresponding Tauc plots presented in the insets of the graphs

UV–vis spectroscopy is an essential tool that is used for the investigation of the optical property of semiconductor nanoparticles [44, 45]. It probes the transition of electrons between the valence band and the conduction band, thereby measures the optical band gap. Although, similar and sometimes used interchangeably, the optical band gap is not necessarily the same as the electronic band gap, which refers to the energy difference between the conduction band maximum (CBM) and the valence band minimum (VBM) [46, 47]. The UV–visible spectra of the green synthesized ZnO, CuO, and ZnO/CuO are shown in Fig. 2. The spectrum of the ZnO NPs (Fig. 2a) showed maximum absorption at approximately 365 nm. A similar peak has been identified in a previous report and indicated the formation of ZnO NPs due to its large excitation binding energy at room temperature [48]. Figure 2b shows the spectrum of CuO NPs with a surface plasmon resonance peak located at 354 nm [49] and a peak around 520 nm [50]. This peak at a higher wavelength may be due to the band edge transition of CuO [51, 52]. The surface plasmon absorption of metal oxide occurs through the collective oscillation of electrons in the free conduction band that is sparked by ambient electromagnetic radiation [53, 54]. Usually, this kind of resonance is observed when the incident light’s wavelength is much greater than its particle diameter [55]. A similar position of the surface plasmon resonance has been reported at 355 nm and 377 nm by Lingaraju, Raja Naika [56] and Ananthalakshmi, Rajarathinam [57] for nanoparticles obtained via the green routes using extracts from *Ruta graveolens* (L.) *and Luffa acutangula* peel. In the spectrum of the nanocomposite (Fig. 2c), the peak due to the surface plasmon resonance broadens out, while the peak ascribed to the band edge emission shifted to 525 nm.

The optical band gap of the nanoparticles was determined from the Tauc’s plot, (Eq. 2) [15, 58] and are presented as insets in the respective absorption spectra.

1 $$\alpha {\text{hv}}\, = C\left( {hv - E_{g} } \right)^{m}$$

Here, α denotes the absorption coefficient, C is a constant, h stands for Planck’s constant, v represents the photon frequency, *Eg* indicates the optical band gap energy, and m is equal to 1/2 for a direct band gap semiconductor. A tangent is drawn on the spectra, and the exact value of the nanoparticles’ band gap energy is obtained by extrapolating the linear region of the plot of (αhv)^2^ on the ordinate axis versus the photon’s energy (*hv*) on the abscissa axis. The optical band gap of the ZnO, CuO nanoparticles and the hybrid ZnO/CuO was obtained as 3.0, 1.51, and 1.65 eV respectively (insets of Fig. 2a, b, and c respectively).

### X-ray diffraction analysis

**Fig. 3:**
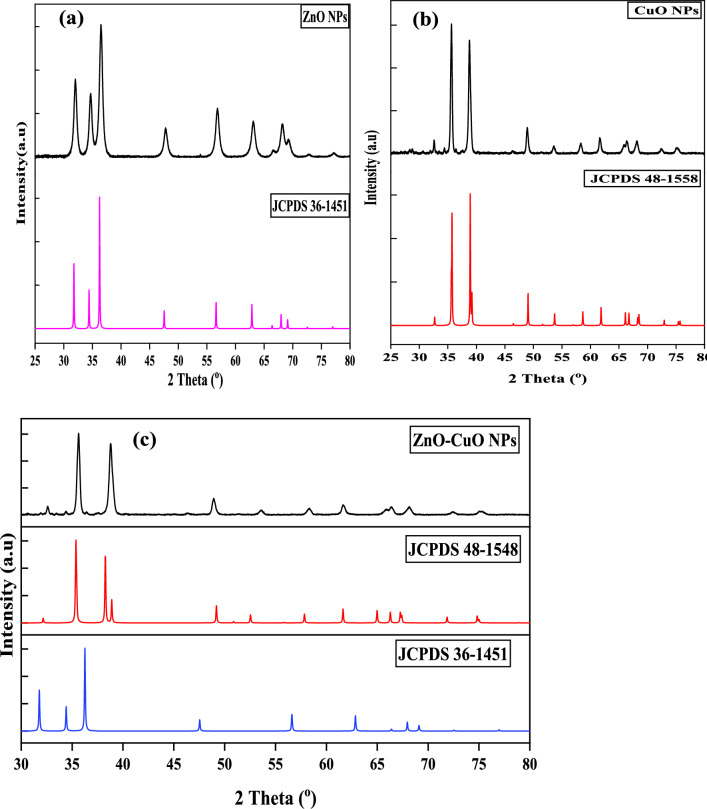
XRD patterns **a** ZnO, **b** CuO, and **c** ZnO/CuO prepared from extract of *P. ostreatus* mushroom substrate

The structural phases and purity of the biosynthesised ZnO, CuO, and ZnO/CuO nanoparticles were confirmed from the XRD patterns, which are presented in Fig. 3. ZnO crystallizes in three forms, namely hexagonal-wurtzite, cubic zincblende, and cubic rock salt [59–61]. Structurally, it has a non-centrosymmetric crystal structure with polar surfaces [62]. In the current study, the diffraction patterns of the ZnO show peaks at 2θ ≈ 32.02°, 34.69°, 36.55°, 47.78°, 56.88°, 63.17°, 66.41°, 68.12°, 73.05°, 77.26° and 81.59^o^ which could be indexed to the reflections from the (100), (002), (101), (102), (110), (103), (112), and (202) crystal planes, respectively, of the hexagonal wurtzite structure (JCP2 card no. 36–1451) Fig. 3a. This is the thermodynamically stable phase of ZnO at ambient conditions [45]. The XRD patterns of the CuO NPs (Fig. 3b) showed diffractions identified at 2θ values of 32.71°, 35.39°, 38.63°, 48.83°, 53.40°, 58.26°, 61.55°, 66.19°, 67.79° and 74.89°, confirming the formation of monoclinic- crystallites structure of CuO, which could be indexed to the (110), (111), (202), (113), (220), (311) and (004) planes respectively of monoclinic phase CuO (JCPDS no. 48–1548). This is also known as the tenorite phase of CuO with a = 4.688, b = 3.422, c = 5.131 A˚ and *β* = 99.50⁰, SG: C_2_/c (no. 15) [63]. No peaks due to Cu or Cu_2_O could be observed which confirmed a complete oxidation of the Cu occurred during the synthesis reaction. The diffraction patterns of the ZnO/CuO nanocomposite showed peaks indicative of the presence of both ZnO and CuO nanoparticles (Fig. 3c). A pronounced reduction in the peak intensity of the ZnO/CuO, relative to the pristine samples, is noticeable and this could be indicative of a reduction in the crystallinity of the sample due to compositing. In addition to crystalline size, lattice strain (ɛ) could also contribute to the reduction in the intensity and broadening of the XRD peak. Lattice strain in nanoparticles is mostly caused by the stress field created by the extra volume of the grain boundaries [64]. Similar to the pristine nanoparticle, no foreign peaks is identifiable in the patterns of the nanocomposite and this confirms the purity of the ZnO/CuO nanocomposite [65]. The crystalline size of the nanomaterial was evaluated from the most intense peak (101) using Debye—Scherer equation [58, 66]. (2).

2 $$D = \frac{KJ}{{\beta hkl}} \cos \theta$$

In this formula, D represents the particle size (in nm), K is Scherrer’s constant (0.90), λ is the X-ray wavelength (0.15406 nm), β is the full width at half maximum (FWHM), and θ is Bragg’s angle of reflection. The average crystallite sizes obtained were 42.4 nm for ZnO, 65.30 nm for CuO, and 55.42 nm for the ZnO/CuO nanocomposite. The particle size of the synthesized nanoparticles is similar to the reported sizes in previous studies [67, 68].

### Morphological analysis

**Fig. 4:**
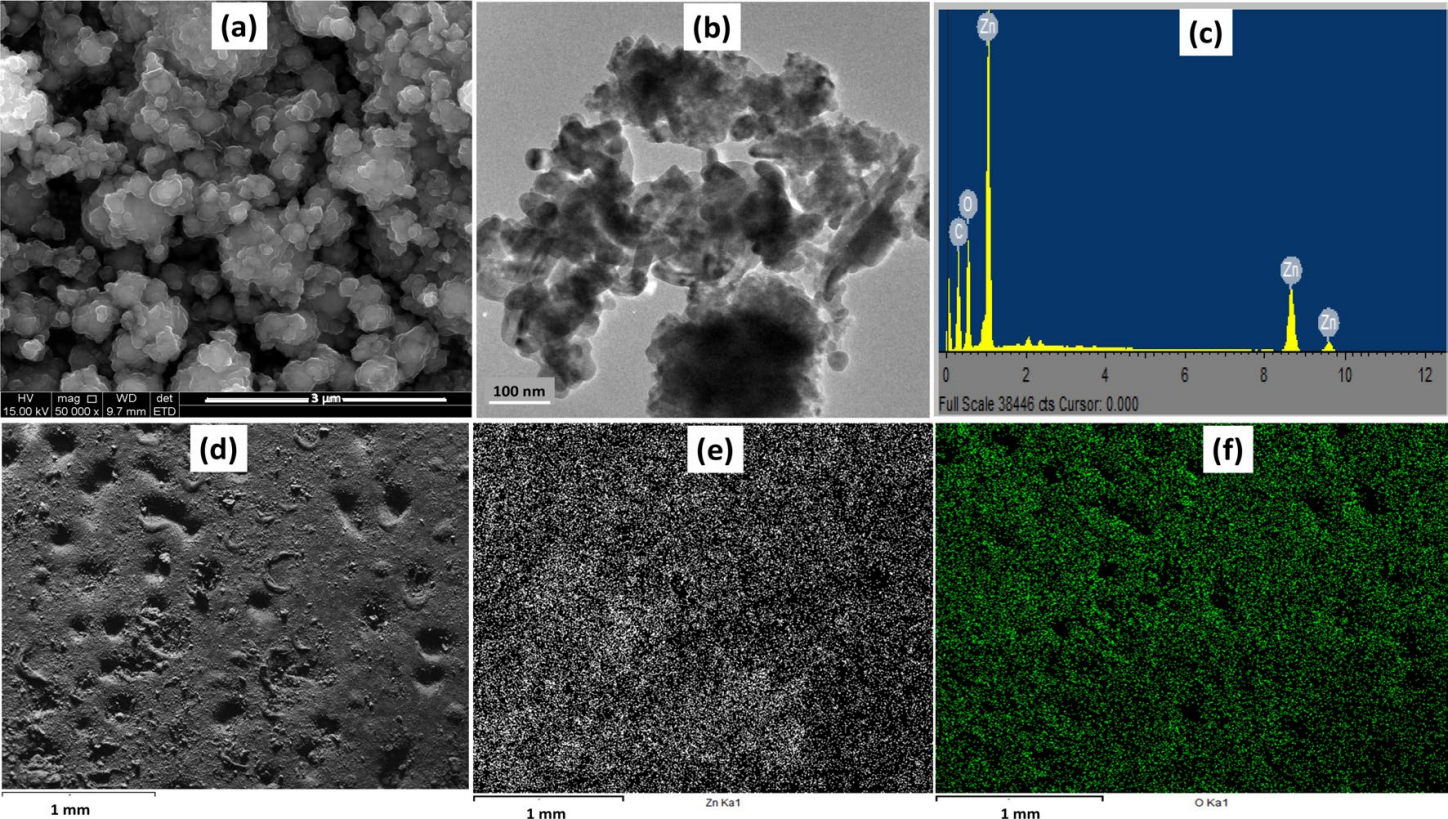
**a** SEM, **b** TEM images and **c** EDAX spectrum showing the composition and **d** Elemental mapping of ZnO depicting **e** Zn and **f** O of the green synthesized ZnO nanoparticles

**Fig. 5:**
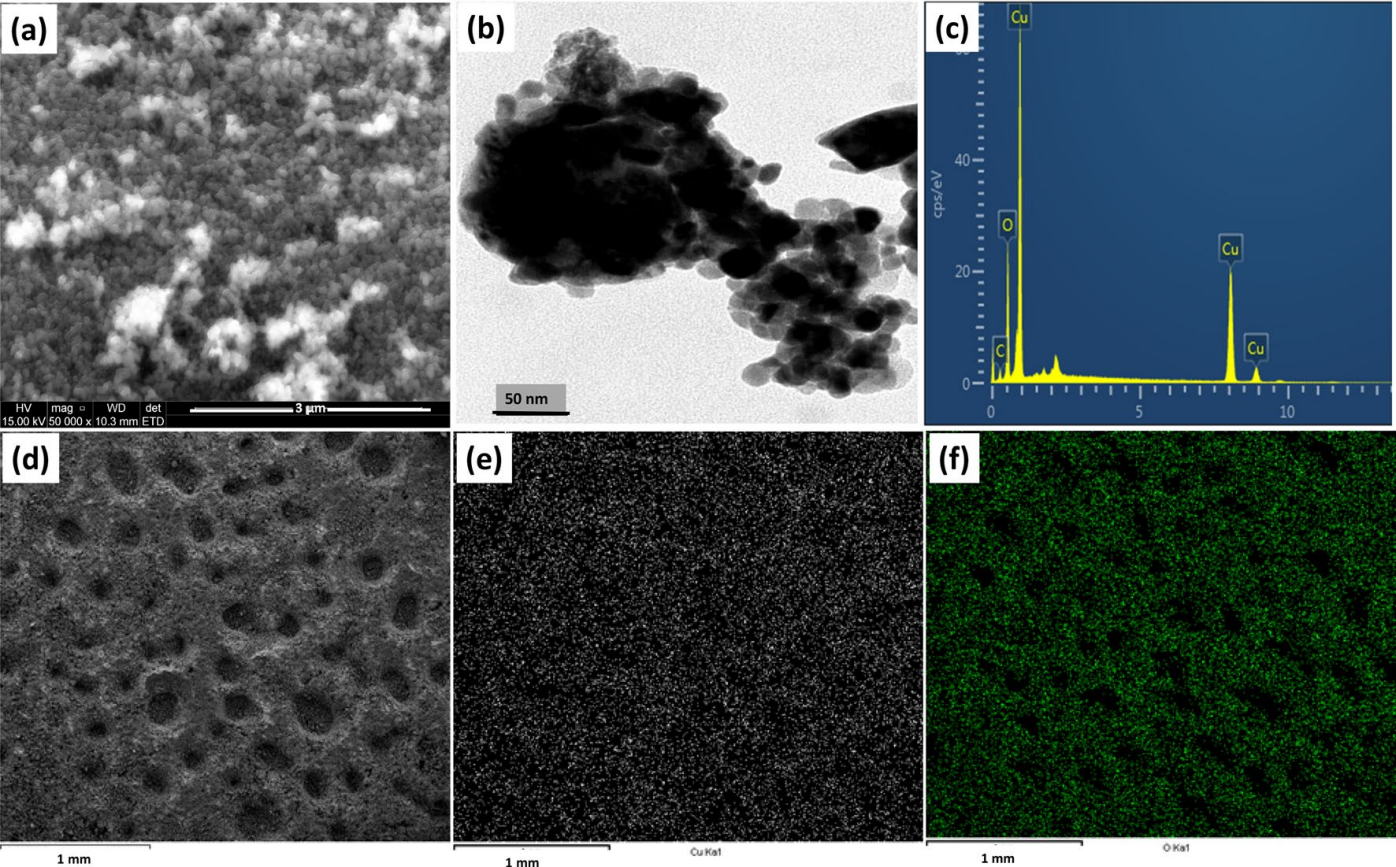
**a** SEM, **b** TEM images and **c** EDAX spectrum showing the composition and **d** Elemental mapping of CuO depicting **e** Cu and **f** O of the green synthesized CuO nanoparticles

SEM analysis was used to study the microstructural properties of ZnO NPs, and Fig. 4a shows the shape of the nanoparticles at a magnification of 3 µm. The image presents a spherically shaped nanoparticles, and a more in-depth exploration of the morphology is shown by the transmission electron microscopy image of Fig. 4b. Here, the particles agglomeration was distinct with no definite edge. The synthesis of ZnO nanoparticles often involve a high temperature calcination, which increases the surface reactivity and promotes agglomeration [69–71]. An EDX measurement was used to determine the elemental composition of the ZnO, and the result presented in Fig. 4c depicts the presence of zinc (Zn) and oxygen (O) as the two major elements in the synthesized ZnO. Figure 5d–f show elemental mapping of the ZnO depicting a uniformly dispersed element of the green synthesized ZnO nanoparticles.

**Fig. 6:**
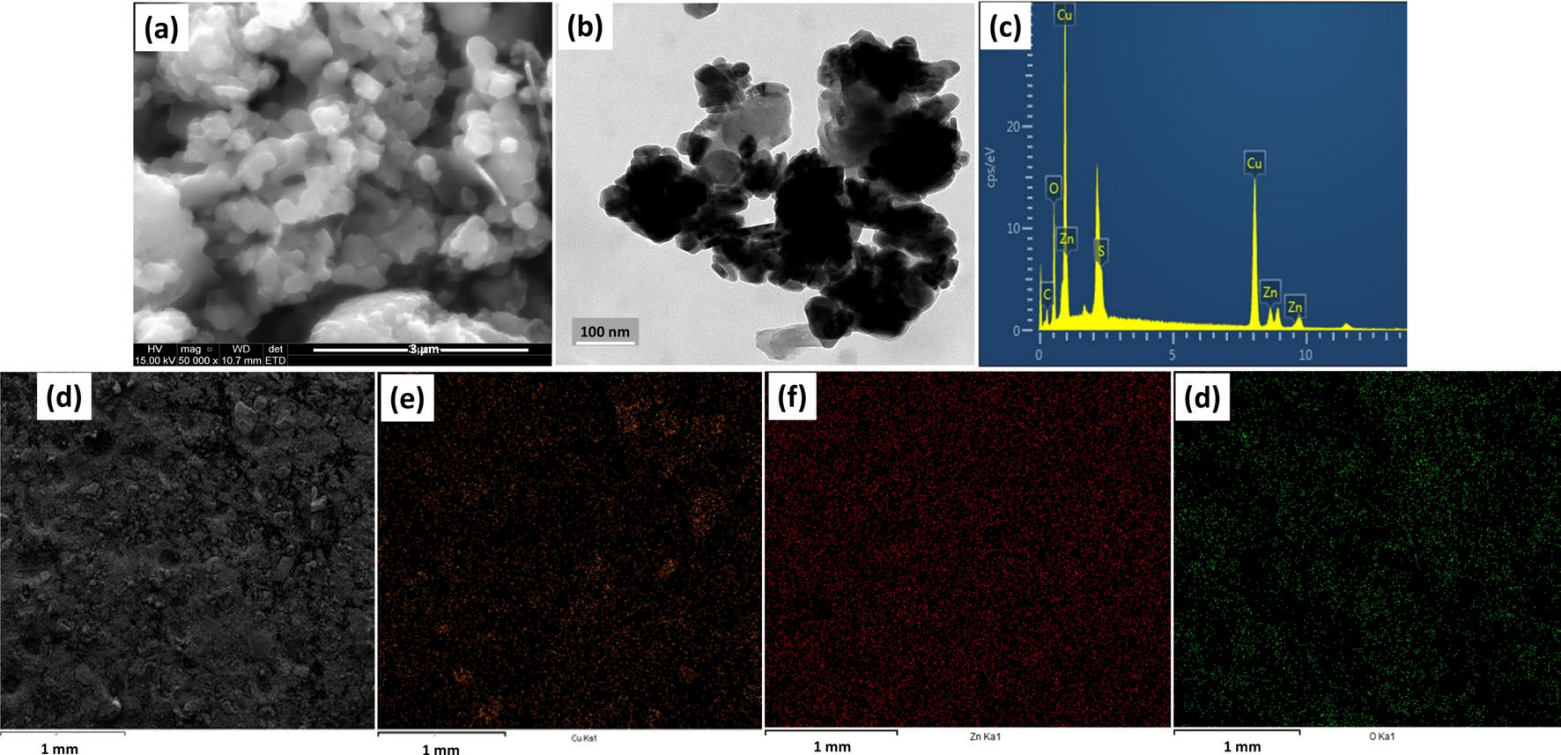
**a** SEM, **b** TEM images, **c** EDAX spectrum showing the composition, and **d** Elemental mapping of ZnO/CuO depicting **e** Zn, **f** Cu, and **g** O of the green synthesized ZnO/CuO nanoparticles

The SEM image of the CuO (Fig. 5a) depicts a tightly packed structure of the nanoparticles, which is corroborated by the TEM image presented in Fig. 5b. The internal morphology shows dense spherical particles and were confirmed to be mainly composed of copper (Cu) and oxygen (O) according to the results of the EDX measurement presented in Fig. 5c. The distribution of the elements across the surface of the particles was explore by the elemental mapping and Figs. 5d–f demonstrates the presence of copper and oxygen on the surface of the nanoparticles. The microarchitecture of the ZnO/CuO powder appears to be a composite of the morphological architecture of both ZnO and CuO nanoparticles. It shows the presence of the spherical morphology as well as the tightly packed structure of nanoparticles (Fig. 6a). The internal structural investigation revealed highly agglomerated particles, including a unique layered structure which may be due to the stacking of particles, Fig. 6b. No distinguishing features or definite shape could be identified due to obvious agglomeration. The elemental analysis obtained from the EDX spectrum (Fig. 6c) and the mapping spectra (Fig. 6d–g) offer vital information about the composite, indicating the presence of Cu, Zn, and O. This information is confirmatory of the successful synthesis of the nanocomposite to form a heterojunction system.

### Biological application

#### Antioxidant activity of NPs

Zinc oxide and copper oxide act as antioxidants by protecting the cellular defence against oxidative stress. Antioxidant activity using the 1,1-diphenyl-2-picrylhydrazyl (DPPH) assay is based on the degree of discoloration of its purple colouration. DPPH solution has a deep violet colour, which changes to pale yellow upon the addition of an antioxidant and the extent of this decolouration indicates the antioxidant capacity [58, 72]. A standard antioxidant drug is often used as a reference or control. The antioxidant process involves an important mechanism that maintains oxidative stress and prevents membrane damage within the cell Manimaran, Balasubramani [73].

The assay involves the scavenging ability of nanoparticles against DPPH, a stable free radical with spare electrons distributed across the molecule. These electrons prevent dimerization, unlike typical free radicals. The nitrogen atom’s odd electron in DPPH is reduced by accepting a hydrogen atom from the nanoparticles, forming hydrazine. This delocalization produces a deep violet color with an absorption peak at ~ 520 nm in ethanol. When DPPH is mixed with nanoparticles capable of donating hydrogen, the violet colour fades, indicating a reduction [74].

**Fig. 7:**
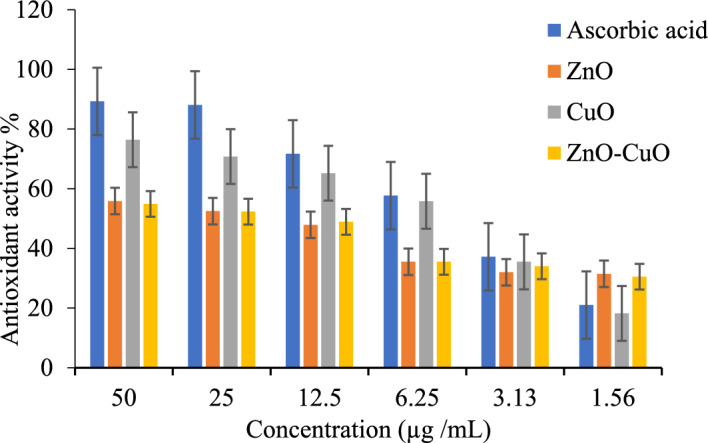
Antioxidant activity (%) of ZnO, CuO and ZnO/CuO hybrid NPs

The percentage inhibition activity of each of these metal oxide nanoparticles was calculated, and the results are summarised in Table 1 and presented in Fig. 7. The IC_50_ of the ZnO, CuO, and ZnO/CuO were obtained as 2.15, 2.16, and 3.18 µg/mL, which were lower than the value obtained for Ascorbic acid (4.25 µg/mL) and indicate a higher antioxidant property of the nanoparticles compared to the standard drug used. This shows that the ascorbic acid was less effective in capturing the free radicals in DPPH compared to the nanoparticles, similar to earlier reports of Elemike et al., [75] and Jobe et al. [76].

**Table 1:** Antioxidant activity (%) of ZnO, CuO and ZnO/CuO hybrid NPs

Test samples	Sample concentrations (µg /mL)						IC50 (µg/mL)
	50	25	12.5	6.25	3.13	1.56	
Ascorbic acid	89.3 ± 22.2	88.1 ± 11.0	71.7 ± 5.4	57.7 ± 2.6	37.2 ± 1.2	21.0 ± 0.4	4.25
ZnO	55.9 ± 0.2	52.5 ± 0.3	47.9 ± 0.2	35.5 ± 0.6	32.0 ± 0.1	31.5 ± 0.2	2.15
CuO	76.4 ± 0.1	70.8 ± 0.1	65.2 ± 0.1	55.8 ± 0.2	35.5 ± 0.1	18.2 ± 0.1	3.18
ZnO/CuO	54.9 ± 0.1	52.3 ± 0.1	48.9 ± 0.01	35.5 ± 0.1	34.0 ± 0.1	30.5 ± 0.04	2.16

Values are reported as mean inhibition% ± standard deviation (n = 3). Ascorbic acid is the standard.

Generally, the intrinsic antioxidant properties of nanoparticles depend on factors such as the size, shape, surface charge, composition of the nanoparticles, as well as the environment. Their high surface area relative to their volume, allows more active sites to interact with reactive oxygen species (ROS) or free radicals [77]. This enhances their ability to neutralize oxidative stress.

In the current study, the nanoparticles were specially selected on the basis that zinc oxide and copper oxide nanoparticles can donate electrons to free radicals, thereby neutralizing them and preventing oxidative chain reactions [78]. In addition, these nanoparticles can release Zn^2+^ and Cu^2+^ ions or active species, which can interact with and neutralize ROS [79]. This reaction also contributes to their antioxidant properties.

#### Cytotoxicity analysis of NPs

**Fig. 8:**
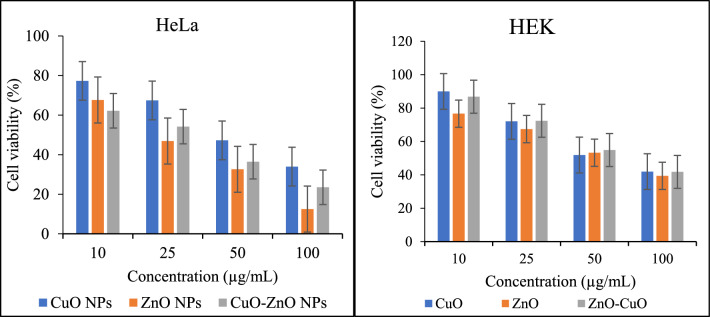
Cytotoxicity activities of the ZnO, CuO and ZnO/CuO NPs

This assay evaluates the reduction of yellow MTT (3-(4,5-dimethylthiazol-2-yl)−2,5-diphenyltetrazolium bromide) to blue formazan by mitochondrial succinate dehydrogenase. Nanoparticles interact with MTT, enabling dehydrogenases in active mitochondria to cleave the tetrazolium ring, indicating the number of viable cells [80]. The assay compares the optical density (ODV) of wells containing drug-incubated cells to those without drug exposure in the context of drug sensitivity assessments. This comparison makes it possible to assess how medications affect the vitality of cells [81]. The drug sensitivity is commonly measured as the drug’s concentration needed to achieve 50% growth inhibition relative to the growth of control cells that have not received treatment [82, 83]. This quantity refers to the 50% inhibitory concentration (IC_50_) [84]. In this study, the results demonstrated that all nanoparticles exhibited strong cytotoxic effects on both types of cells. ZnO NPs showed the highest activity, with IC_50_ values of 1.94 μM for HEK 293 cells and 3.23 μM for HeLa cells, outperforming CuO and ZnO/CuO NPs (see Table 2 and Fig. 8). Additionally, the assay results revealed that cell viability for both HEK 293 and HeLa cells decreased as the concentration of the nanoparticles increased. Similar results have been reported in previous studies involving ZnO NPs [58, 85].

**Table 2:** Cytotoxicity activities of the ZnO, CuO and ZnO/CuO NPs

Cell line	Test sample	Sample concentrations ((µg/mL)				IC50
		10	25	50	100	
HeLa	CuO	77.28 ± 0.04	67.42 ± 0.05	47.25 ± 0.03	34.00 ± 0.03	2.93
	ZnO	67.65 ± 0.04	46.89 ± 0.03	32.64 ± 0.04	12.53 ± 0.04	1.94
	ZnO/CuO	62.19 ± 0.02	54.19 ± 0.03	36.46 ± 0.04	23.52 ± 0.05	2.06
HEK	CuO	89.99 ± 0.02	72.07 ± 0.06	51.87 ± 0.08	41.95 ± 0.07	3.35
	ZnO	76.64 ± 0.02	67.44 ± 0.04	53.27 ± 0.04	39.44 ± 0.04	3.23
	ZnO/CuO	86.85 ± 0.05	72.37 ± 0.08	54.87 ± 0.04	41.78 ± 0.06	3.41

Values are reported as mean cell viability (%), ± standard deviation (n = 3).

Cancerous cells’ uptake by nanoparticles typically occurs through endocytosis, a cellular process where these cells engulf the nanoparticles. This cellular process can be passive, whereby it is driven by the physicochemical properties of the nanoparticles, or active, facilitated by surface modifications that target specific receptors overexpressed on cancer cells. Tumor cells often exhibit enhanced endocytic activity due to their high metabolic demand and altered membrane dynamics. Common pathways include clathrin-mediated endocytosis, caveolae-mediated endocytosis, macropinocytosis, or phagocytosis, depending on the nanoparticle size, shape, surface charge, and functionalization. Upon internalization, the ZnO, CuO, and ZnO/CuO nanoparticles were transported through endosomal pathways, where they may escape lysosomal degradation or release their therapeutic payload in a controlled manner, thereby exerting their anticancer effects [86–88].

## Conclusion

In this study, ZnO and CuO nanoparticles and the composite of these metal oxides (ZnO/CuO) were successfully synthesized through a simple and eco-friendly route. This method utilizes the phytocomponent of the spent *P. ostreatus* mushroom substrate to mediate the formation of the metal oxide nanoparticles. It offered several advantages including a simple, non-toxic, low-energy, and cost-effective route to nano-synthesis. Characterization technique such as XRD analysis was used to establish hexagonal and monoclinic crystalline phases for the ZnO and CuO NPs respectively, while UV–vis analysis showed absorption in the ultraviolet region of the solar spectrum with peaks centered at 354 (ZnO) and 365 nm (CuO). The morphologies and sizes were established using SEM and TEM analyse, while the EDS spectral analysis indicated the elemental composition of the metal oxides. All the nanoparticles exhibited dose-dependent antioxidant activities better than the Ascorbic acid used as control, and the anticancer assay conducted using the MTT methods demonstrated that ZnO NPs exhibited the best inhibitory activities against the HEK 293 and HeLa cells compared to the CuO and ZnO/CuO NPs. Hence, the protocol provides facile and large-scale techniques for the synthesis of ZnO, CuO, and ZnO/CuO NPs that could be anticipated as good for biomedical applications.

## Data Availability

“Data is provided within the manuscript”.
